# Regulation of I_Ks_ Potassium Current by Isoproterenol in Adult Cardiomyocytes Requires Type 9 Adenylyl Cyclase

**DOI:** 10.3390/cells8090981

**Published:** 2019-08-27

**Authors:** Yong Li, Thomas Hof, Tanya A. Baldwin, Lei Chen, Robert S. Kass, Carmen W. Dessauer

**Affiliations:** 1 Department of Integrative Biology and Pharmacology, McGovern Medical School, University of Texas Health Science Center, Houston, TX 77030, USA; 2 Department of Pharmacology, Department of Medicine, College of Physicians and Surgeons of Columbia University, New York, NY 10032, USA

**Keywords:** adenylyl cyclase, AC9, A-kinase anchoring protein, AKAP, cyclic AMP, cardiomyocyte, Yotiao, KCNQ1, protein kinase A, potassium channels

## Abstract

The subunits KCNQ1 and KCNE1 generate the slowly activating, delayed rectifier potassium current, I_Ks_, that responds to sympathetic stimulation and is critical for human cardiac repolarization. The A-kinase anchoring protein Yotiao facilitates macromolecular complex formation between I_Ks_ and protein kinase A (PKA) to regulate phosphorylation of KCNQ1 and I_Ks_ currents following beta-adrenergic stimulation. We have previously shown that adenylyl cyclase Type 9 (AC9) is associated with a KCNQ1-Yotiao-PKA complex and facilitates isoproterenol-stimulated phosphorylation of KCNQ1 in an immortalized cell line. However, requirement for AC9 in sympathetic control of I_Ks_ in the heart was unknown. Using a transgenic mouse strain expressing the KCNQ1-KCNE1 subunits of I_Ks_, we show that AC9 is the only adenylyl cyclase (AC) isoform associated with the KCNQ1-KCNE1-Yotiao complex in the heart. Deletion of AC9 resulted in the loss of isoproterenol-stimulated KCNQ1 phosphorylation in vivo, even though AC9 represents less than 3% of total cardiac AC activity. Importantly, a significant reduction of isoproterenol-stimulated I_Ks_ currents was also observed in adult cardiomyocytes from I_Ks_-expressing AC9KO mice. AC9 and Yotiao co-localize with N-cadherin, a marker of intercalated disks and cell–cell junctions, in neonatal and adult cardiomyocytes, respectively. In conclusion, AC9 is necessary for sympathetic regulation of PKA phosphorylation of KCNQ1 in vivo and for functional regulation of I_Ks_ in adult cardiomyocytes.

## 1. Introduction

The slow delayed rectifier (I_Ks_) current in cardiomyocytes (CMs) is produced by a potassium channel composed of four KCNQ1 (Kv7.1) α-subunits along with the accessory KCNE1 (MinK) β-subunits [1,2]. In the heart, this outward potassium channel is important for the repolarization late phase of the cardiac action potential and becomes more critical as sympathetic activity increases the heart rate [3]. Stimulation of beta-adrenergic receptors leads to an increase in cAMP production by adenylyl cyclase, activation of protein kinase A (PKA), and a subsequent increase in the I_Ks_ current and a shortening of the cardiac action potential duration. The mechanism underlying the enhanced I_Ks_ current is due to direct protein kinase A (PKA) phosphorylation of the N-terminal residues S27 and S92 of KCNQ1, causing the channel to open more quickly and more often when in the presence of KCNE1 and an intact microtubule network [4,5,6,7]. PKA phosphorylation of KCNQ1 is dependent on its association with the A-kinase anchoring protein Yotiao (AKAP9). Yotiao positions KCNQ1 near not only to PKA, but also to termination signals such as phosphodiesterase PDE4D3 and the phosphatase PP1 [5,8].

Genetic mutations in KCNQ1 that affect trafficking, assembly, or regulation prolong the cardiac action potential duration, lengthening the time between the start of the Q wave to end of the T wave (QT interval) of the electrocardiogram [9,10,11,12]. Long QT syndrome (LQT) is a hereditary cardiac condition associated with life-threatening arrhythmias and sudden death. Of particular note are human mutations within KCNQ1 (LQT1) or Yotiao (LQT11) that disrupt the Yotiao-KCNQ1 macromolecular complex, block KCNQ1 phosphorylation, and are at the greatest risk of lethal arrhythmias in the face of sympathetic stimulation [5,13]. Disruption of the interface between KCNQ1 and KCNE1 (LQT5, P127T) also suppresses Yotiao-mediated PKA phosphorylation of KCNQ1 and cAMP-dependent stimulation of I_Ks_ [14]. Thus, local signalosomes consisting of KCNQ1 and its regulators are critical for proper sympathetic control of cardiac repolarization.

Multiple isoforms of adenylyl cyclase (AC) are expressed in the heart that respond to beta-adrenergic stimulation [15,16]. Several cardiac AKAPs, including AKAP5, mAKAP, D-AKAP2, and Yotiao, anchor a subset of AC isoforms to potentially generate local pools of cAMP [17,18,19,20,21,22]. We have previously shown that AC9 forms complexes with KCNQ1 and Yotiao in mouse and guinea pig heart [18,23]. Moreover, KCNQ1 phosphorylation is facilitated by AC9 when co-expressed with Yotiao in Chinese hamster ovary (CHO) cells [23]. However, AC9 represents less than 3% of the total AC activity in the heart [24], therefore, it is unknown whether AC9 is actually required to regulate I_Ks_ in CMs. Using a transgenic mouse strain that expresses the subunits of human I_Ks_, KCNQ1-KCNE1, we show that knockout of AC9 results in a complete loss of Yotiao- and KCNQ1-associated AC activity. Isoproterenol injection in vivo results in a 60% increase in KCNQ1 phosphorylation, which is abolished in mice lacking AC9. This is consistent with decreased isoproterenol-stimulated I_Ks_ current in adult CMs from AC9 deficient mice. Viral expression of a catalytically inactive mutant of AC9 blocks isoproterenol-stimulated phosphorylation of KCNQ1 in CHO cells stably expressing the channel. KCNQ1 and beta2-adrenergic receptors show co-localization at the sarcolemma and intercalated disks of adult CMs. AC9 and Yotiao co-localize with N-cadherin, a marker of intercalated disks and cell-cell junctions, in neonatal and adult CMs, respectively. Therefore, AC9 is necessary for sympathetic regulation of PKA phosphorylation of KCNQ1 in vivo and for functional regulation of I_Ks_ in adult CMs and is likely a necessary component of the KCNQ1-KCNE1-Yotiao-PKA complex in the human heart that regulates cardiac repolarization.

## 2. Materials and Methods

### 2.1. Generation of IKs-AC9KO Gene-Targeted Mice

The AC9KO mouse strain, B6; 129S5-Adcy9Gt(neo)159Lex/Mmucd, identification number 011682-UCD, was obtained from the Mutant Mouse Regional Resource Center, a National Institute of Health-funded strain repository (originally donated to the MMRRC by Lexicon Genetics, Woodlands, TX, USA). The AC9KO strain was derived and backcrossed on C57BL6/J (WT) as described [24]. AC9KO mice were crossed with transgenic mice (TG^+^) expressing hKCNQ1-hKCNE1 under the control of the α-MHC-promoter for cardiac-specific expression (from Charles River Laboratories and referred to as MK24 in [5]; designated IKs mice herein). The hKCNQ1-hKCNE1 TG^+^ mice express functional slow delayed rectifier potassium channel currents (I_Ks_) normally absent from murine cardiac ventricular myocytes. Mice were genotyped by polymerase chain reaction (PCR) analysis with primers specific for KCNQ1 and AC9. All animal protocols were approved by the Institutional Animal Care and Use Committee (IACUC) at the University of Texas Health Science Center at Houston in accordance with the Animal Welfare Act and NIH guidelines (Animal Welfare Assurance # A3413-01).

### 2.2. Plasmids and Adenoviruses

A Flag-tag (MDYKDDDDK) plus two residue linker (GA) was inserted in frame of the N-terminus of human AC9 using nested PCR primers. The resulting clone was sequenced, and the activity of the tagged protein verified upon expression in HEK293 cells and Sf9 cells. To create a catalytically inactive AC9, aspartate 399 was mutated to alanine using QuikChange II Site-Directed Mutagenesis Kit (Agilent Technologies, Santa Clara, CA, USA). For adenoviral expression, Flag-tagged catalytically inactive AC9 (AC9-D399A, AC9d) was inserted into the Kpn I/XbaI restriction sites of pShuttle-CMV vector. Recombinant adenoviruses were produced according to the manufacturer’s instructions (AdEasy Adenoviral Vector Systems, Agilent Technologies). Appropriate clones were selected by RT-PCR and sequenced.

### 2.3. Cell Culture and Transfections

CHO cell line that stably expresses hKCNQ1-hKCNE1 was described previously [13]. The cell line was maintained in a Ham’s F12 culture media with 10% fetal bovine serum that contains Hygromycin B (500 µg/mL) in a 37 °C incubator with 5% CO_2_ and transfected with the indicated plasmids using Lipofectamine 2000 (ThermoFisher Scientific, Waltham, MA, USA). For analysis of KCNQ1 phosphorylation, cells were transfected with pCDNA3 or myc-tagged Yotiao for 24 h and then infected with green fluorescent protein (GFP) or AC9d adenoviruses for an additional 40 hours. Isoproterenol stocks were prepared in 0.1 mM ascorbic acid and 1 mM thiourea, pH 7. Isoproterenol was diluted in Ham’s F12 media (no serum) immediately prior to treatments. For cAMP accumulation assays, cells were treated as indicated and reactions were stopped with 0.1N HCl. Cyclic AMP was quantitated by enzyme immunoassay (Assay Designs).

### 2.4. Antibodies Used for Immunoprecipitation and Western Blotting

Antibodies used for immunoprecipitation (IP) and western blotting (WB) include anti-β-actin (C4, Santa Cruz Biotechnology, Santa Cruz, CA, USA), goat anti-cyclase IX (N-18, Santa Cruz Biotechnology, Santa Cruz, CA, USA), and normal mouse or rabbit IgG (Santa Cruz Biotechnology, Santa Cruz, CA, USA). A phospho-S27 KCNQ1 antibody was raised as described previously [25]. A commercial KCNQ1 antibody (C20) (Santa Cruz Biotechnology, Santa Cruz, CA, USA) was used to detect total KCNQ1. The rabbit anti-Yotiao antibody was generated and characterized as described [18]. For immunostaining, see Table 1 and methods below.

For analysis of PKA phosphorylation in the heart, IKs and IKs-AC9KO mice were injected with saline or isoproterenol (2 μg/g body weight, IP). Animals were sacrificed 5 min later and heart tissue was harvested. Cardiac extracts were prepared in the presence of phosphatase inhibitors. Equal protein supernatants were subjected to western blot analysis as described in figure legends.

### 2.5. Adenylyl Cyclase Activity and IP-AC Assays

Preparation of heart extracts and measurement of AC activity after immunoprecipitation (IP-AC assay) were performed as previously described [19,23,26]. Cardiac membranes were prepared from 6–8 weeks old mice. AC activity was stimulated with 300 nM purified GTPγS-Gαs and cAMP was detected by enzyme immunoassay (Assay Designs) or using [α^32^P]ATP [27].

### 2.6. Adult Cardiomyocyte Isolation

After mouse euthanasia, the heart was rapidly removed and mounted on a Langendorff apparatus and perfused retrogradely with a HEPES-based isolation buffer containing (in mmol/L): 112 NaCl, 5.4 KCl, 1.7 NaH_2_PO_4_, 1.6 MgCl_2_, 20.4 HEPES, 30 Taurine, 2 DL-carnitine, 2.3 creatine, 5.4 glucose, 10 2,3-Butanedione monoxime (pH 7.2 with NaOH). When the coronary circulation had cleared of blood, the heart was perfused for 8 min with isolation buffer containing 0.1 mg/mL liberase (MilliporeSigma, Burlington, MA, USA) and 20 µM CaCl_2_. The ventricles were then excised from the heart, placed in the HEPES-based solution containing 0.2% of bovine serum albumin (BSA) and 20 µM CaCl_2_ and minced. Ventricles were dissociated by triturating and allowed to sediment. The supernatant, containing isolated cells was removed and procedure was repeated until ventricular dissociation was complete. After sedimentation, cells were suspended and stored in the HEPES-based solution containing 1 mM CaCl_2_.

### 2.7. Single Cell Electrophysiology

Mouse ventricular cardiomyocytes (CMs) were placed on the stage of an inverted microscope (Nikon, Tokyo, Japan) and currents were recorded at room temperature (23–25 °C) using the whole-cell configuration of the patch-clamp technique. An Axopatch 200B (Molecular Devices, San Jose, CA, USA) amplifier, controlled by a Pentium PC connected via a Digidata 14440A A/D converter (Molecular Devices, San Jose, CA, USA), was used for data acquisition and analysis using pClamp software (Molecular Devices, San Jose, CA, USA). Patch pipettes were made from Type 8250 glass (King Precision Glass, Claremont, CA, USA) and resistance was typically 0.9–1.6 MΩ when filled with intracellular solution.

For I_Ks_ recording, pipette solution contained (in mmol/L): 110 K-aspartate, 5 ATP-K_2_, 11 EGTA, 10 HEPES, 5.5 CaCl_2_, 1 MgCl_2_ and perfusion solution contained (in mmol/L): 132 NaCl, 4.8 KCl, 1.2 MgCl_2_, 2 CaCl_2_, 10 HEPES, 5 glucose (pH 7.4 with NaOH). To stimulate β-adrenergic receptors, 1 µM isoproterenol (MilliporeSigma, Burlington, MA, USA) and 1 µM okadaic acid potassium salt (MilliporeSigma, Burlington, MA, USA) were concomitantly added to the perfusion solution.

The holding potential for cells was kept at −40 mV for all experiments. To trigger I_Ks_ current, depolarizing steps of 2 secs to 60 mV were applied every 15 sec. Tail current were then elicited by repolarizing the cell to −40 mV. Maximum tail current amplitude and current amplitude at the end of the 2 secs pulse were measured. Voltage dependence of activation, determined from amplitudes of deactivating current tails normalized to maximum tail current plotted vs. test pulse voltage, were fitted with a Boltzmann equation to determine channels mid-point of activation (V_50_).

### 2.8. Neonatal Cardiomyocyte Isolation and Adenoviral Infection

Neonatal rat CMs were isolated as previously described with minor modifications [28]. Briefly, hearts from 1–3 days old Sprague-Dawley rats were excised, atria removed, and ventricles minced. Ventricles underwent a series of five digestions by agitation with collagenase Type II and pancreatin; cells from each digestion were pooled. Cardiomyocytes were enriched via percoll gradient centrifugation and plated on fibronectin coated glass bottom MatTek dishes (P35G-1.5-14-C) or 6-well plates in complete media (40% DMEM, 40% HAMS/F10, 20% FBS, 1% P/S). 24–48 h later cells were washed with phosphate buffered saline (PBS) to remove debris and media was replaced with DMEM + 2% FBS + 1% P/S. Cells were infected 96–120 h post isolation with adenoviruses (multiplicity of infection 50–100) for 60 h.

### 2.9. Cardiomyocyte Immunocytochemistry

Neonatal cardiomocytes were fixed with 2% PFA in PBS for 15 min, permeabilized with 0.1% Trition X 100 for 10 min, blocked with 5% goat serum in PBS and incubated with primary antibody overnight at 4 °C. Primary and secondary antibodies were diluted in: 1X PBS + 0.075% Triton X-100 + 5% goat serum (Table 1). After digestion was complete for adult CMs [29], cells were collected via centrifugation, reintroduced to calcium stepwise, fixed with 100% ice cold ethanol, and stored at −20 °C until used for immunocytochemistry. Ethanol was removed by washes with PBS + 5% goat serum (3 times, 5 min each). Cells were then incubated with primary antibody (Table 1) overnight at 4 °C. All images were acquired on a Nikon A1R confocal with a 60X oil, numerical aperture 1.49 objective.

### 2.10. Statistical Analysis.

Data are expressed as mean ± standard deviation (SD), except where noted. Differences between samples were determined using analysis of variance (ANOVA) followed by multiple comparison tests between groups, or unpaired t-test for comparison between two groups, as indicated in each figure legend. Significant p values are indicated as follows: (*) denotes a *p* value < 0.05, (**) < 0.01 and (***) < 0.001. All analyses were performed using SigmaPlot statistical analysis software (Systat Software, Inc., San Jose, CA, USA).

## 3. Results

### 3.1. Genetic Ablation of AC9 Results in Preweaning Subviability

AC9 association with Yotiao-KCNQ1 facilitates KCNQ1 phosphorylation by PKA in CHO cells stably expressing KCNQ1-KCNE1 [23]. To investigate the in vivo function of AC9 regulation of I_Ks_ in the heart, we used a mouse gene-trap deletion of AC9 [24]. However, a functional I_Ks_ is largely absent in adult mice, therefore we crossed the AC9 deletion with a transgenic strain containing cardiac-specific expression of hKCNQ1-hKCNE1 (designated IKs herein) [5]. KCNQ1-KCNE1 TG^+^/AC9^−/−^ (IKs-AC9KO) mice showed no distinctive phenotypes, except for a preweaning subviability that was previously reported for the AC9KO strain [24].

### 3.2. Deletion of AC9 Results in Loss of Yotiao- and KCNQ1-Associated AC Activity but no Alterations in Total Cardiac AC Activity

**Figure 1:**
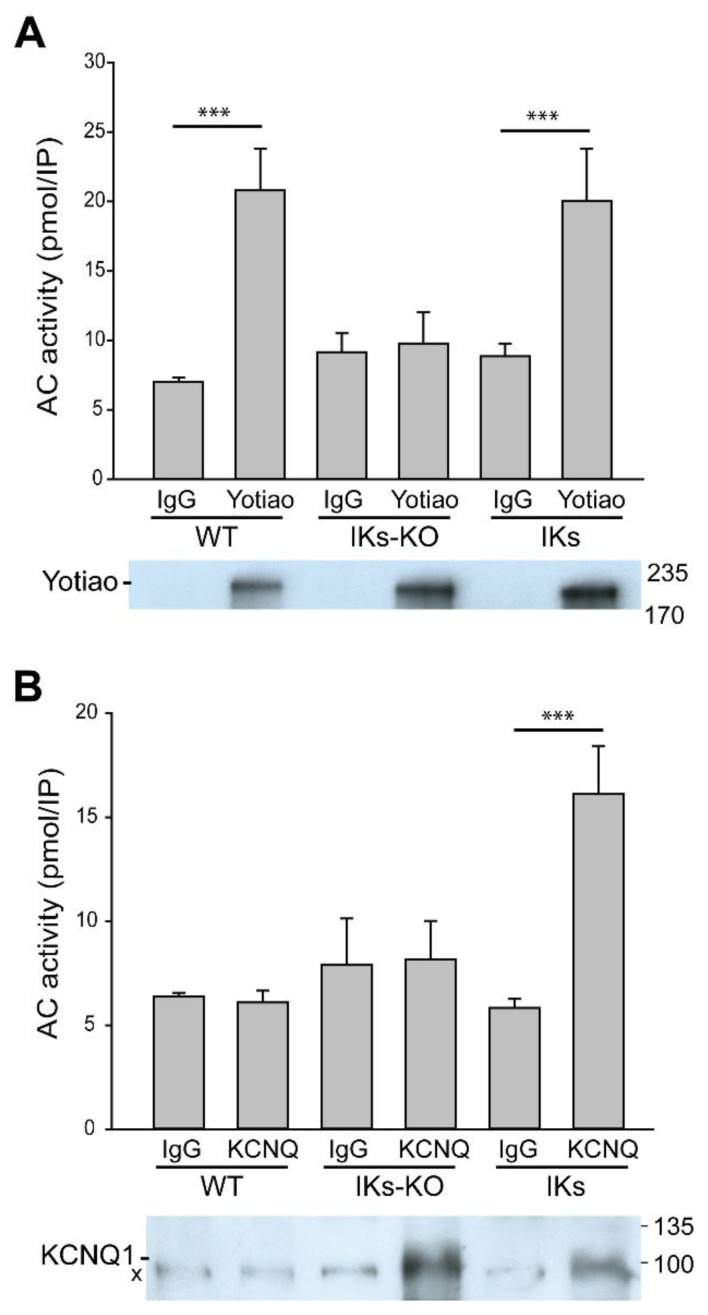
AC9 association with Yotiao and KCNQ1. Heart extracts from WT, IKs or IKs-AC9KO mice were subjected to immunoprecipitation (IP) with IgG (rabbit) or anti-Yotiao (**A**) and goat IgG or anti-KCNQ1 (**B**). The resulting immunoprecipitate was stimulated with 300 nM Gαs to measure AC activity. Data are shown as mean +/− SD. A portion of the IP’s from each sample was subjected to WB analysis for Yotiao (**A**) or KCNQ1 (**B**) and are shown below. Statistics: Kruskal-Wallis Analysis of Variance followed by Dunn’s or Bonferroni’s comparison method. n = 6–8, *** *p* < 0.001.

To detect AC9, we examined association of AC activity with specific macromolecular complexes [23,24]. Heart extracts were subjected to immunoprecipitation with antibodies against Yotiao, the KCNQ1 subunit of I_Ks_, or the corresponding IgG (Figure 1A, B) and the amount of AC activity in the resulting immunoprecipitate was measured by addition of exogenous activated Gαs (termed IP-AC assay) [19,26]. Significant associated AC activity was pulled down with Yotiao from WT and IKs hearts but not IKs-AC9KO heart (Figure 1A), indicating that AC9 is the only AC associated with Yotiao in IKs mice, as reported for WT [24]. Immunoprecipitation of KCNQ1 from WT hearts showed no detectable AC activity or KCNQ1 protein in western blots compared to IgG controls, consistent with reports of negligible I_Ks_ currents in adult WT mice (Figure 1B) [5,30,31]. Significant AC activity was associated with KCNQ1 in the heart from IKs mice, but not IKs-AC9KO mice when compared to control IgG samples, suggesting that AC9 is also the only AC isoform associated with the KCNQ1-Yotiao complex.

**Figure 2:**
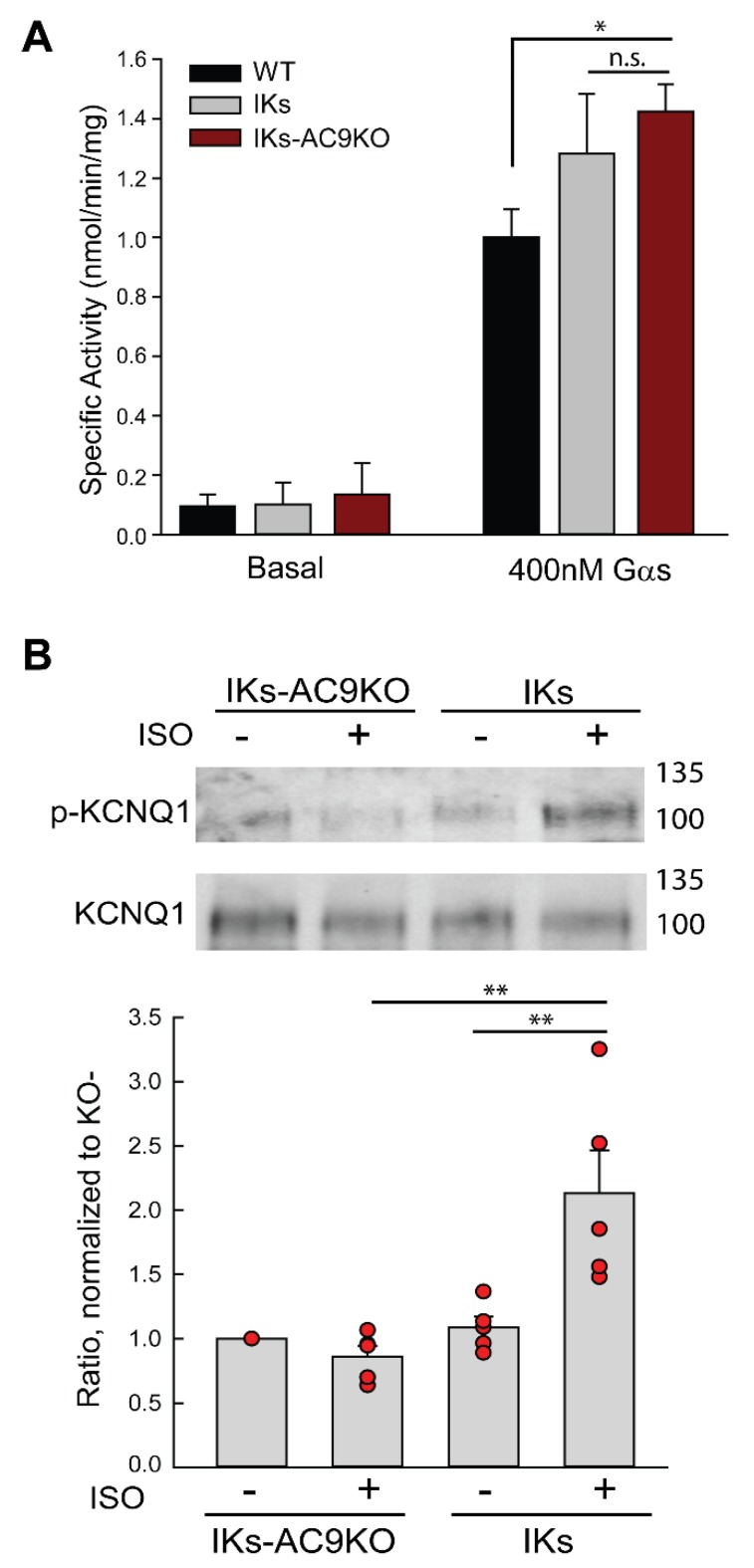
AC9 is required for sympathetic stimulation of KCNQ1 phosphorylation in vivo. Membranes were prepared from IKs-WT versus IKs-AC9KO heart. (**A**) AC activity was measured under basal conditions and upon stimulation with 300 nM Gαs (n = 3, performed in duplicate or triplicate). Data are shown as mean +/− SD. Statistics: One Way Analysis of Variance followed by Tukey comparison method for Gαs-stimulated AC activity; * *p* < 0.05 for WT compared to IKs and IKs-AC9KO. (**B**) IKs and IKs-AC9KO mice were injected with saline or isoproterenol (2 μg/g body weight, IP). Animals were sacrificed 5 min later and heart tissue was harvested. Cardiac extracts were prepared in the presence of phosphatase inhibitors. Equal protein supernatants were subjected to WB analysis; the ratio of phosphoprotein to total KCNQ1 was quantitated and normalized to IKs-AC9KO control. Data are shown as mean +/− SEM, with individual experimental data points in red. Statistics: Two-way Analysis of Variance followed by a Bonferroni multiple comparison method. n = 5, ** *p* < 0.001.

Global basal and Gαs-stimulated cardiac AC activity were similar for membranes isolated from IKs and IKs-AC9KO heart, while Gαs-stimulated AC activity was slightly increased in IKs-AC9KO compared to WT (Figure 2A). This is consistent with our previous findings that AC9 represents less than 3% of total cardiac AC activity [24], and thus a reduction in global AC activity was not predicted. To determine if the low level of cAMP production by AC9 in the heart was sufficient for sympathetic regulation of KCNQ1 phosphorylation, intraperitoneal injection of saline or isoproterenol in IKs and IKs-AC9KO mice was used to evaluate changes in the PKA phosphorylation of serine 27 of KCNQ1. Heart tissue was harvested 5 min post injection and cardiac extracts were subjected to immunoprecipitation with anti-KCNQ1 antibodies. Isoproterenol injection resulted in a two-fold increase in KCNQ1 S27 phosphorylation, which was absent in hearts from IKs-AC9KO mice (Figure 2B). These data indicate that AC9 is required for PKA-mediated phosphorylation of KCNQ1 in response to isoproterenol stimulation in vivo.

### 3.3. Loss of AC9 Reduces β-Adrenergic Regulation of I_Ks_ Currents in Adult Cardiomyocytes

**Figure 3:**
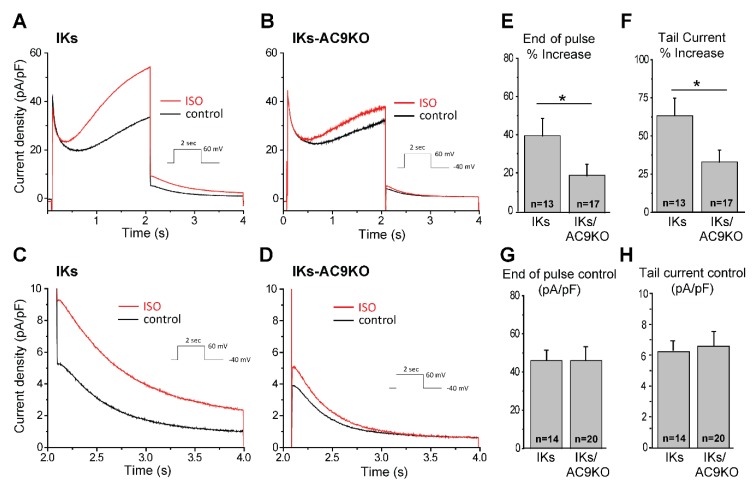
Deletion of AC9 dampens isoproterenol stimulation of I_Ks_. Representative traces of I_Ks_ recordings in ventricular myocytes isolated from IKs (**A**) and IKs-AC9KO mice (**B**) before (control) and after perfusion of isoproterenal and okadaic acid (ISO). Enlargement of I_Ks_ tail current in ventricular myocytes isolated from IKs (**C**) and IKs-AC9KO mice (**D**) before (control, black line) and after perfusion of isoproterenol and okadaic acid (ISO, red line). Increase in I_Ks_ current at the end of pulse at + 60 mV (**E**) and in the tail current (**F**) in myocytes from IKs and IKs-AC9KO in control and after 1 µM isoproterenol and 1 µM okadaic acid. Total I_Ks_ density at the end of the 2 sec pulse at + 60 mV (**G**) and I_Ks_ tail density (**H**) in IKs and IKs-AC9KO ventricular myocytes was unchanged. Statistics: t-test, * *p* < 0.05, (Mann-Whitney), number of cells is indicated on the figure for each condition.

Sympathetic stimulation of I_Ks_ currents is known to be a key mechanism of β-adrenergic-induced action potential shortening as heart rate increases. PKA phosphorylation of serine 27 and 92 of KCNQ1 shifts the voltage dependence of activation to more negative potentials, increases the rate of activation of I_Ks_ and decreases the rate of deactivation to ultimately increase the open probability of the channel to accelerate repolarization [32,33,34]. To evaluate the requirement of AC9 to this mechanism, we compared the I_Ks_ response to β-adrenergic stimulation in adult mouse cardiomyocytes (CMs) from mice expressing KCNQ1-KCNE1 (IKs), without and with AC9 deletion (IKs-AC9KO). CMs from IKs and IKs-AC9KO mice presented the typical I_Ks_ current and tail current tracings (Figure 3A–D). The rate of current decay, gives rise to the so-called “tail current” and is indicative of the I_Ks_ channel closing, while the maximum amplitude of the tail current, immediately following the end of the test pulse, is proportional to the number of open channels (Figure 3). Total current density was similar between IKs and IKs-AC9KO myocytes (46 ± 5 vs. 46 ± 7 pA/pF respectively for end of pulse; 6.2 ± 0.7 vs. 6.5 ± 1 pA/pF respectively for tail current) (Figure 3G and H). Treatment of CMs from IKs mice with cAMP and the protein phosphatase 1 inhibitor okadaic acid (1 µM) significantly increases I_Ks_ currents [5]. Similarly, β-adrenergic stimulation of I_Ks_ with isoproterenol and okadaic acid increased I_Ks_ currents in CMs with endogenous AC9 present; however this response was significantly blunted in IKs-AC9KO myocytes (39 ± 9 vs. 19 ± 6% increase for end of pulse for IKs and IKs-AC9KO, respectively; 62 ± 12 vs. 33 ± 7% increase of tail current) (Figure 3E and F).

**Figure 4:**
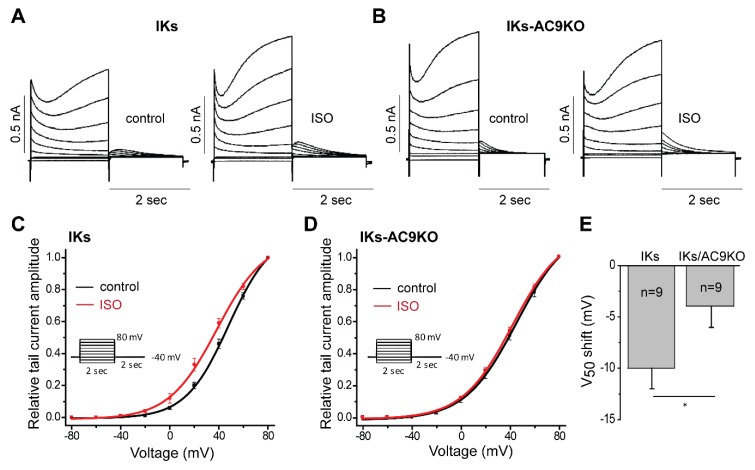
AC9 deletion impairs isoproterenol-induced regulation of I_Ks_. I_Ks_ currents in ventricular myocytes of IKs (**A**) and IKs-AC9KO (**B**) mice in before (control, left panels) and after perfusion of 1 µM isoproterenol and 1 µM okadaic acid (ISO, right panels). Currents were elicited by applying depolarizing potentials to various levels ranging from −80 to +80 mV for 2 s from a holding potential of −40 mV. I-V relationships for tail current in IKs (**C**) and IKs-AC9KO (**D**) before (control) and after 1 µM isoproterenol and 1 µM okadaic acid (ISO). (**E**) Shift of V_50_ induced by 1 µM isoproterenol and 1 µM okadaic acid perfusion of myocytes from IKs and IKs-AC9KO mice. Statistics: t-test, * *p* <0.05, (Mann-Whitney), number of cells is indicated on the figure for each condition.

The effect of β-adrenergic stimulation on I_Ks_ voltage dependence of activation was also investigated. Representative traces, recorded in control and after ISO + okadaic acid perfusion, with various depolarizing pulses are shown for CMs from IKs and IKS-AC9KO mice (Figure 4A,B). Deactivating tail current amplitudes normalized to maximum tail current amplitude were plotted with respect to the pulse voltage (Figure 4C,D). Interestingly, the voltage giving rise to the half-maximal activation (V_50_) was not significantly different between IKs-WT and IKs-AC9KO under control conditions (43 ± 2 vs. 42 ± 1 mV respectively). However, the isoproterenol-induced shift of the voltage dependence of activation was significantly larger in CMs with endogenous AC9 present compared to IKs-AC9KO myocytes (−10 ± 2 mV vs. −4 ± 2 mV respectively) (Figure 4C–E). These data show a key role for AC9 in the β-adrenergic regulation of I_Ks_ currents in adult CMs.

### 3.4. Expression of Catalytically Inactive AC9 Blocks KCNQ1 Phosphorylation

**Figure 5:**
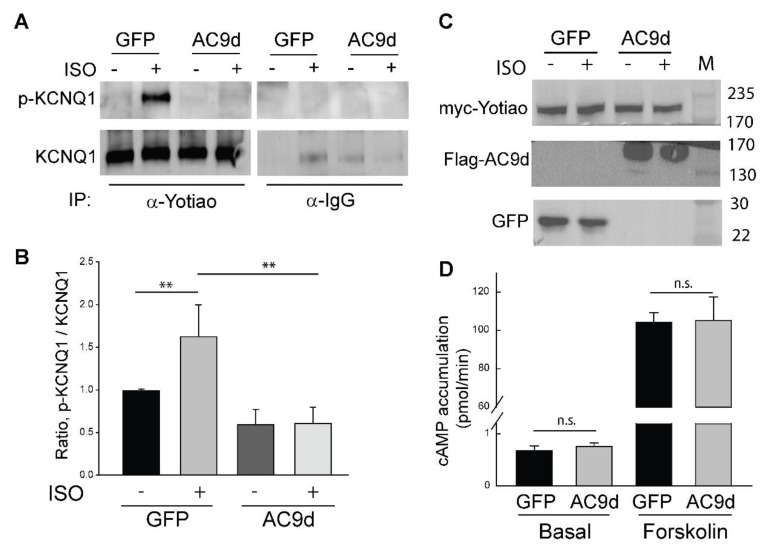
Expression of catalytically inactive AC9 decreases isoproterenol-stimulated phosphorylation of KCNQ1. CHO cells stably expressing KCNQ1 were transfected with myc-tagged Yotiao for 24 h prior to infection with GFP control or catalytically inactive AC9-D399A (AC9d) adenoviruses for an additional 40 hours. Cells were treated with vehicle or isoproterenol (1 μM) for 5 min prior to cell lysis. The cell lysate was subjected to immunoprecipitation (IP) with IgG (rabbit) or anti-Yotiao. (**A**) The ratio of p-KCNQ1 to total KCNQ1 was measured by WB; quantitation is shown in (**B**). #, ** *p* < 0.05 t-test, n = 3, mean +/− SD. (**C**) Total cell lysates were probed for expression of myc-Yotiao, Flag-AC9d, and GFP. (**D**) CHO cells were transfected and infected as in (**A**) and treated for 5 min with 1 mM 3-isobutyl-1-methylxanthine (Basal) +/− 20 µM forskolin. cAMP was quantitated by enzyme immunoassay.

Binding of AC9 to the Yotiao-KCNQ1 complex should regulate local cAMP production and subsequent KCNQ1 phosphorylation. If this were the case, displacing AC9 with a catalytically inactive enzyme would lead to reduced KCNQ1 phosphorylation. Mutation of D399 to alanine deletes a key metal-binding residue in the active site of AC9, reducing Gαs-stimulated activity by >90% [24]. We examined CHO cells stably expressing KCNQ1 with transient expression of Yotiao. After infection and adenoviral expression of AC9-D399A for 48 h, cells were stimulated with 1 µM isoproterenol for 5 min and then assayed by immunoprecipitation of Yotiao followed by western blotting for PKA phosphorylation of Ser-27 of KCNQ1 versus total KCNQ1 (Figure 5). Isoproterenol treatment of GFP-infected cells elicited a 62% increase in KCNQ1 phosphorylation, whereas adenoviral expression of AC9-D399A eliminated any effect of isoproterenol on KCNQ1 phosphorylation (Figure 5). The effect of AC9d expression was not due to non-specific effects on other endogenous AC isoforms, as expression of AC9d had no effect on either basal or forskolin-stimulated AC activity (Figure 5D). Competing endogenous AC9 with an inactive enzyme was sufficient to block beta-agonist stimulated phosphorylation of KCNQ1.

### 3.5. Subcellular Distribution of AC9 and Yotiao in Cardiomyocytes

**Figure 6:**
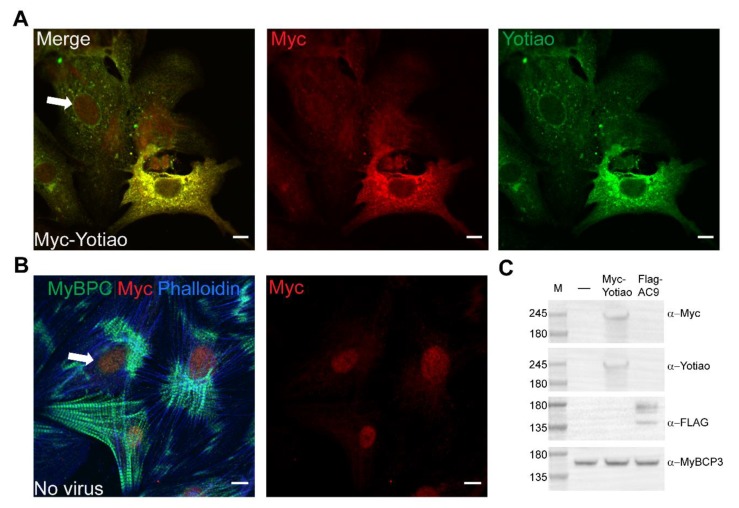
Validation of Yotiao Antibody. (**A**) Neonatal rat CMs were infected with Myc-Yotiao for 60 h and stained with Myc (red) and Yotiao (green). Separate channels are shown to the right of the composite. (**B**) Control cells were stained with myosin binding protein C 3 (MyBPC) (green), Myc (red), and phalloidin (blue). A separate image of Myc staining is shown to the right of the composite image. Note, non-specific nuclear staining of Myc (arrows). Scale bar represents 10 µm (**A**,**B**). Images are representative of >10 CMs per condition from ≥2 isolations. (**C**) Equal protein lysates from neonatal rat CMs were subjected to western blot analysis with anti-Myc, Yotiao, FLAG, and MyBPC3. M (marker).

In mouse and guinea pig ventricular CMs KCNQ1 localizes to intercalated discs, sarcolemma, and transverse tubules [6,33]. Although never verified, subcellular localization of Yotiao and AC9 had been assumed to be similar. To directly evaluate subcellular localization of Yotiao, myc-tagged Yotiao was expressed in neonatal rat ventricular CMs. The overlap of immunostaining using anti-Myc and anti-Yotiao antibodies in CMs expressing myc-tagged Yotiao was used to evaluate antibodies for use in detecting endogenous Yotiao (Figure 6A). Cells expressing Myc-Yotiao had a Pearson’s correlation coefficient of 0.91 +/− 0.04 for Myc and Yotiao antibodies, whereas in uninfected cells the Pearson’s correlation coefficient was only 0.62 +/− 0.08. Uninfected cells showed non-specific nuclear staining of Myc (Figure 6B). Western blotting of neonatal rat CMs expressing Myc-Yotiao and Flag-AC9 confirms expression (Figure 6C). Overexpression of Yotiao was not used for further analysis of subcellular localization or to quantitate co-localization with AC9 as adenoviral expression was cytotoxic and often did not result in proper localization.

**Figure 7:**
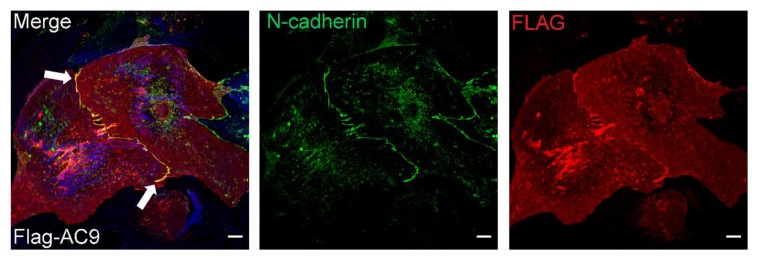
AC9 co-localizes with N-cadherin at the plasma membrane and points of cell–cell contacts in neonatal rat cardiomyocytes. Neonatal rat CMs were infected with Flag-AC9d for 60 h then stained with N-cadherin (green), FLAG (red), and phalloidin (blue). Arrows point to co-localization of AC9 with N-cadherin at the plasma membrane or points of cell–cell contact. Individual channels are shown to the right of composite images. Scale bar represents 10 µm, n > 10 cells per condition from ≥2 CM isolations.

**Figure 8:**
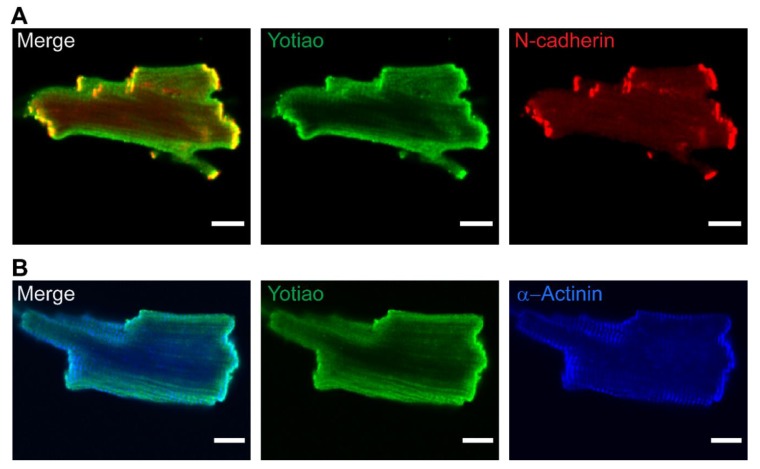
Endogenous Yotiao localizes to sarcolemmal membrane and intercalated discs in adult mouse cardiomyocytes. Wild-type adult mouse CMs were stained with Yotiao (green) and (**A**) N-cadherin (red) or (**B**) α-actinin (blue). Individual channels are shown to the right of composite images. Scale bar represents 10 µm, n > 10 CMs per antibody condition.

Next, to evaluate subcellular distribution of AC9, Flag-AC9d was expressed in neonatal CMs. AC9 appeared diffusely throughout the cell but notably concentrated at the sarcolemma and points of cell–cell contact, co-localizing with N-cadherin (Figure 7). Immunostaining with anti-Flag was only observed in Flag-AC9 infected cells, with no background staining observed in surrounding CMs. Available antibodies (commercial and newly generated) against AC9 were tested; however, no specific staining of overexpressed or endogenous AC9 was detected in neonatal or adult CMs, using cells from AC9KO mice as controls. While endogenous AC9 localization in adult CMs could not be directly investigated, we would predict it localizes to the sarcolemma and intercalated discs in adult CMs. Endogenous expression of Yotiao in neonatal rat CMs showed diffuse staining throughout the cell (Figure 6A). However, immunostaining of Yotiao in adult mouse ventricular CMs showed subcellular distribution to the sarcolemma and intercalated discs (Figure 8A,B). Diffuse Yotiao staining was also noted throughout adult CMs with a striated patterning that largely did not overlap with alpha-actinin (Figure 8B). No nuclear localization of Yotiao was observed in adult CMs. Strong overlap of Yotiao with N-cadherin supports localization at intercalated discs (Figure 8A), consistent with patterns observed for KCNQ1 in adult CMs [6,33] and AC9 in neonatal CMs (Figure 7).

## 4. Discussion

The I_Ks_ channel is well known as the major outward repolarizing K^+^ current during cardiac action potentials, particularly upon sympathetic stimulation. Approximately 40–50% of all long QT syndrome patients carry a mutation in the I_Ks_ subunit, KCNQ1, which puts them at risk of potentially life-threatening arrhythmia. Impaired PKA regulation of I_Ks_ increases the risk of arrhythmia in these patients, particularly during rigorous exercise [35]. The formation of a complex consisting of I_Ks_ and the scaffolding protein Yotiao serves to co-localize PKA near the channel to facilitate PKA phosphorylation of KCNQ1 and enhance channel activity [5,34]. However efficient PKA activation also requires a nearby source of cAMP. Anchoring of AC to an AKAP scaffold can lead to the generation of local pools of cAMP [21,22], and can sensitize bound PKA substrates to the effects of cAMP by up to 3 orders of magnitude [36]. Yotiao binds AC isoforms 1, 2, 3, and 9, but of these, only AC9 is present in adult CMs [23]. Previous work showed that expression of both Yotiao and AC9 enhanced PKA phosphorylation of KCNQ1 in CHO cells [23], but the necessity of these interactions for PKA phosphorylation of KCNQ1 or I_Ks_ activity in CMs was unknown. We now show that AC9 is a critical component of the Yotiao-I_Ks_ complex for proper sympathetic regulation of the channel.

### 4.1. Association of IKs with AC9

Using a transgenic mouse line expressing KCNQ1-KCNE1 in the heart, we showed that both Yotiao- and KCNQ1-associated AC activity in the heart were completely lost upon deletion of AC9, indicating that AC9 is the only AC isoform associated with the Yotiao-I_Ks_ complex. Deletion of AC9 does not alter ribonucleic acid (RNA) levels for any other AC isoform in the heart (except for a 35% decrease of AC3), nor does it alter AKAP79/150-associated AC5 and AC6 activity [24]. Moreover, basal and Gαs-stimulated activity in cardiac membranes from IKs-AC9KO mice were unchanged as compared to IKs mice. This is not surprising as AC9 represents only a small fraction (<3%) of total AC activity in the heart. Yet despite the small pool of cAMP generated by AC9, its deletion significantly blunted isoproterenol stimulation of KCNQ1 phosphorylation in the heart. PKA phosphorylation of KCNQ1 increases the open probability of I_Ks_ and shifts the voltage dependence for activation of the channel. However, this enhancement of I_Ks_ currents by isoproterenol was significantly diminished in adult CMs from IKs-AC9KO mice. Deletion of AC9 showed no alterations in global cardiac PKA phosphorylation upon isoproterenol injection in AC9KO vs. WT mice [24]. To further show a requirement for AC9, a catalytically inactive form of AC9 was used (AC9d). Competition of endogenous AC9 with the dominant negative AC9d was sufficient to abolish isoproterenol-stimulated KCNQ1 phosphorylation in CHO cells stably expressing KCNQ1-KCNE1. Importantly, expression of AC9d did not alter overall basal or forskolin-stimulated activity.

Previous studies have shown co-localization of KCNQ1 and beta2-adrenergic receptors at the sarcolemma and intercalated disks of adult CMs, while in mouse and guinea pig ventricular CMs KCNQ1 can also be found on transverse tubules [6,33]. In the current study, AC9 and Yotiao were found to co-localize with N-cadherin, a marker of intercalated disks, in neonatal and adult CMs, respectively. However, AC9 localization could only be detected upon adenoviral overexpression of the enzyme. The very low level of AC9 expression in the heart prevented detection of the endogenous enzyme. Localization of Yotiao also presents challenges. All antibodies that recognize the 250 kD splice variant of AKAP9 (commonly referred to as Yotiao) also recognize the 350 and 450 kD splice variants of AKAP9. These longer forms are often found within the cytoplasm, at the Golgi apparatus and on microtubules and centrosomes [37], making co-localization studies of endogenous Yotiao and AC9 difficult.

### 4.2. AC9 Regulation and Physiology

AC9 is the most divergent of the nine transmembrane AC isoforms and shows little forskolin stimulation, except upon conditional stimulation with Gαs [38]. Compared to the major cardiac isoforms AC5/6, AC9 has decreased sensitivity to Gαs, emphasizing the requirement for anchoring of AC9 close to receptors and downstream PKA substrates. Also, AC9 is not inhibited by Gαi, consistent with a lack of I_Ks_ regulation by Gαi/o-coupled muscarinic receptors [39]. As discussed above, loss of AC9 has no measurable effects on global AC or PKA stimulated activities. Yet, similar to the regulation of I_Ks_, local cAMP production by AC9 is critical for baseline PKA phosphorylation of Hsp20 [24]. The loss of this cardioprotective signal may give rise to the decreased early filling velocity during diastolic relaxation observed in AC9KO mice. A similar phenotype is observed in patients with long QT syndrome (LQT1 and LQT2) [40].

## 5. Conclusions

AKAPs scaffolding of upstream and downstream components facilitate signal propagation from adenylyl cyclase activation and cAMP production to PKA phosphorylation of anchored substrates. Disruption of AC association within these complexes can have effects in numerous systems. For example, disruption of AC anchoring to mAKAP can promote cardiomyocyte hypertrophy [19], while disruption of AC binding to AKAP79/150 dampens isoproterenol regulation of glutamate receptors [41], prostaglandin regulation of TRPV1 [36], and the spontaneous activity of nociceptors after spinal cord injury [42]. In the heart, sympathetic regulation of I_Ks_ is no exception, requiring Yotiao to coordinate the localization of small local pools of cAMP generated AC9 with the activation of PKA and phosphorylation of KCNQ1 to enhance I_Ks_ currents.

## Figures and Tables

**Table 1:** Antibody dilutions and product information.

Antibody	Company/Product Number	Dilution
rabbit anti-Yotiao	Previously described [18]	1:75
mouse anti-DYKDDDDK tag (9A3)	Cell Signaling Technologies (8146)	1:750
mouse anti-Alpha-actinin (sarcomeric)	Sigma (A7811)	1:1000
rabbit anti-Myosin binding protein C 3	Santa Cruz Biotechnology (sc 67353)	1:200
mouse anti-N-cadherin (13A9) DyLight550	Novus Biological (48309R)	1:500
rabbit anti-N-cadherin	Abcam (Ab18203)	1:250
mouse anti-myc tag	Invitrogen (MA1-21316)	1:150
Alexa 647 Phalloidin	Invitrogen (A22287)	1:1000
Alexa 488 anti-rabbit	Invitrogen (A21206)	1:500
Alexa 568 anti-mouse	Invitrogen (A10037)	1:500
Alexa 647 anti-mouse	Invitrogen (A31571)	1:500
